# A Case Series and Brief Review of Literature on Encapsulating Peritoneal Sclerosis: Unveiling the Cocoon

**DOI:** 10.7759/cureus.73802

**Published:** 2024-11-16

**Authors:** Bribin Bright, Roshna Salam, Srikanth Moorthy

**Affiliations:** 1 Radiodiagnosis, Amrita Institute of Medical Sciences, Kochi, IND

**Keywords:** abdominal cocoon, encapsulating peritoneal sclerosis, encapsulating sclerosing peritonitis, idiopathic sclerosing peritonitis, ldlt complications, peritoneal fibrosis, peritoneal sclerosis, sclerosing encapsulating peritonitis (sep), small bowel occlusion, trilaminar appearance

## Abstract

This case series explores four distinct instances of encapsulating peritoneal sclerosis (EPS), a rare but serious condition characterized by the encapsulation of abdominal viscera, commonly referred to as abdominal cocoon. EPS is associated with severe complications, including bowel obstruction and sepsis, which can significantly impact patient outcomes.

The first case involves a 41-year-old male patient who had undergone a liver transplant and ultimately succumbed to extensively drug-resistant (XDR) sepsis. The second case features a 31-year-old male patient diagnosed with abdominal tuberculosis, who successfully recovered following comprehensive antitubercular therapy (ATT). The third case presents a 26-day-old neonate with CHARGE syndrome (coloboma of the eye, heart defects, atresia of the choanae, retardation of growth and development, genital abnormalities, and ear anomalies, including deafness), who tragically succumbed to septic shock. The last case is of a 41-year-old male patient with disseminated tuberculosis who showed marked improvement with appropriate treatment.

These cases illustrate the diverse clinical backgrounds and grave outcomes associated with EPS, highlighting the urgent need for early diagnosis and intervention. Despite EPS being a leading cause of small bowel obstruction in many inpatient settings, its diagnosis is frequently overlooked due to insufficient awareness among healthcare professionals.

This series aims to enhance understanding of the causes, imaging characteristics, and management strategies for EPS. By disseminating this knowledge, we hope to facilitate earlier identification of the condition, particularly through primary imaging techniques such as ultrasound (USG). Ultimately, increasing awareness and understanding of EPS is crucial to improving patient outcomes and reducing the associated morbidity and mortality.

## Introduction

Encapsulating peritoneal sclerosis (EPS), otherwise known as abdominal cocoon, is an inflammatory disease of the peritoneum, characterized by the encapsulation of the abdominal viscera and intestines within a thickened fibrocollagenous membrane [1]. Early detection is crucial for preventing severe complications such as recurrent bowel obstruction, adhesions, perforation, and sepsis, improving prognosis, and expanding treatment options. EPS frequently occurs in patients undergoing peritoneal dialysis for end-stage renal disease [2]. Chronic exposure to dialysate solution during peritoneal dialysis can lead to peritoneal inflammation and scarring, which may progress to peritoneal sclerosis. EPS can also develop following various surgeries and organ transplantations, including liver transplantation, due to trauma and inflammation. The etiology can be a wide spectrum [3-22]. In developing countries, tuberculosis is a common infectious cause; other infectious etiologies include intraperitoneal infections, particularly from *Staphylococcus aureus*,* Pseudomonas*, and various fungal species, which can significantly contribute to the development of EPS, especially in immunocompromised patients. Diagnostic imaging, particularly ultrasound (USG) and computed tomography (CT), plays a critical role in the early identification of EPS.

Peritoneal Abnormalities

The inflammatory process affects the peritoneum diffusely, resulting in widespread peritoneal fibrosis. This manifests as peritoneal thickening, which can be smooth or irregular and nodular.

Small Bowel Abnormalities

The sclerosed, thickened peritoneum surrounds the small bowel loops, leading to bowel obstruction. Varying lengths of small bowel may be tightly enclosed within pockets of fibrotic peritoneum, described as an "abdominal cocoon."

In this case series, we present a comprehensive analysis of four distinct cases of encapsulating peritoneal sclerosis (EPS). Our aim is to illustrate the varied clinical presentations and outcomes of EPS to enhance understanding and aid in diagnosis and early detection. After completing this journal activity, participants will be able to examine the terminology and underlying causes of encapsulating peritoneal sclerosis (EPS), identify the multimodality imaging appearances and complications of EPS, and discuss the differential considerations and management of EPS.

## Case presentation

This case series demonstrates the diverse presentations and outcomes of encapsulating peritoneal sclerosis (EPS) in patients with varying underlying conditions, including post-liver transplant complications, abdominal tuberculosis, congenital syndromic anomalies, and disseminated infections. EPS diagnosis was confirmed through characteristic imaging findings, such as encapsulated ascites, clumped bowel loops, and unique appearances (e.g., "cauliflower" and "concertina-like") observed on contrast-enhanced computed tomography (CECT) and USG. Table 1 demonstrates a summary of four cases, illustrating the varying presentations, imaging features, diagnoses, and outcomes of encapsulating peritoneal sclerosis (EPS) across diverse underlying conditions.

**Table 1:** Case series overview: key data and findings LDLT: living donor liver transplant, CHARGE: coloboma of the eye, heart defects, atresia of the choanae, retardation of growth and development, genital abnormalities, and ear anomalies, including deafness, CT: computed tomography, CECT: contrast-enhanced computed tomography, EPS: encapsulating peritoneal sclerosis, USG: ultrasound, XDR: extensive drug-resistant, TB: tuberculosis, MTB: *Mycobacterium tuberculosis*, RIF: rifampicin. MODS: multiple organ dysfunction syndrome, ATT: antitubercular therapy

	Case 1	Case 2	Case 3	Case 4
History	41-year-old male patient post-LDLT	31-year-old male patient	26-day-old female syndromic neonate with CHARGE syndrome	41-year-old male patient with disseminated tuberculosis
Presentation	Generalized body pain, abdominal distension, occasional difficulty breathing, pedal edema	Abdominal distension, weight loss	Respiratory distress, umbilical cord swelling, post-umbilical hernia repair, bowel anastomosis	Fever, cough, loss of appetite, weight loss
Key imaging feature	CECT showed encapsulated fluid collections (greater sac and lesser sac, small bowels clumped toward the root of the mesentery) with enhancing encapsulation, features of EPS	USG showed central clumping of the small bowel with a "cauliflower" appearance; CECT revealed encapsulated ascites, abdominal cocoon, mesenteric adenopathy, omental nodularities	Initial USG was normal; serial USG and CT showed clumped small bowel loops at the root of the mesentery, concertina-like appearance with enhancing encapsulation, loculated fluid collections, features of EPS	USG and CECT showed the supracolic compartment encapsulated small bowel loops, hyperattenuating ascites with septations, omental caking, multiple nodes, granulomatous lesions in the liver and spleen
Diagnosis	Post-LDLT XDR sepsis with EPS	Abdominal TB with EPS (omental biopsy and GeneXpert MTB-RIF test confirmed)	Post-surgery, congenital shunts, perforation, sepsis with EPS	Wet-type abdominal tuberculosis with EPS
Outcome	Developed XDR sepsis and MODS, resulting in death	Recovered well, discharged on ATT with regular follow-up	Developed septic shock and fecal peritonitis, leading to cardiopulmonary arrest and death	Started on ATT, recovered well, continued ATT with regular follow-up

Case 1

**Figure 1:**
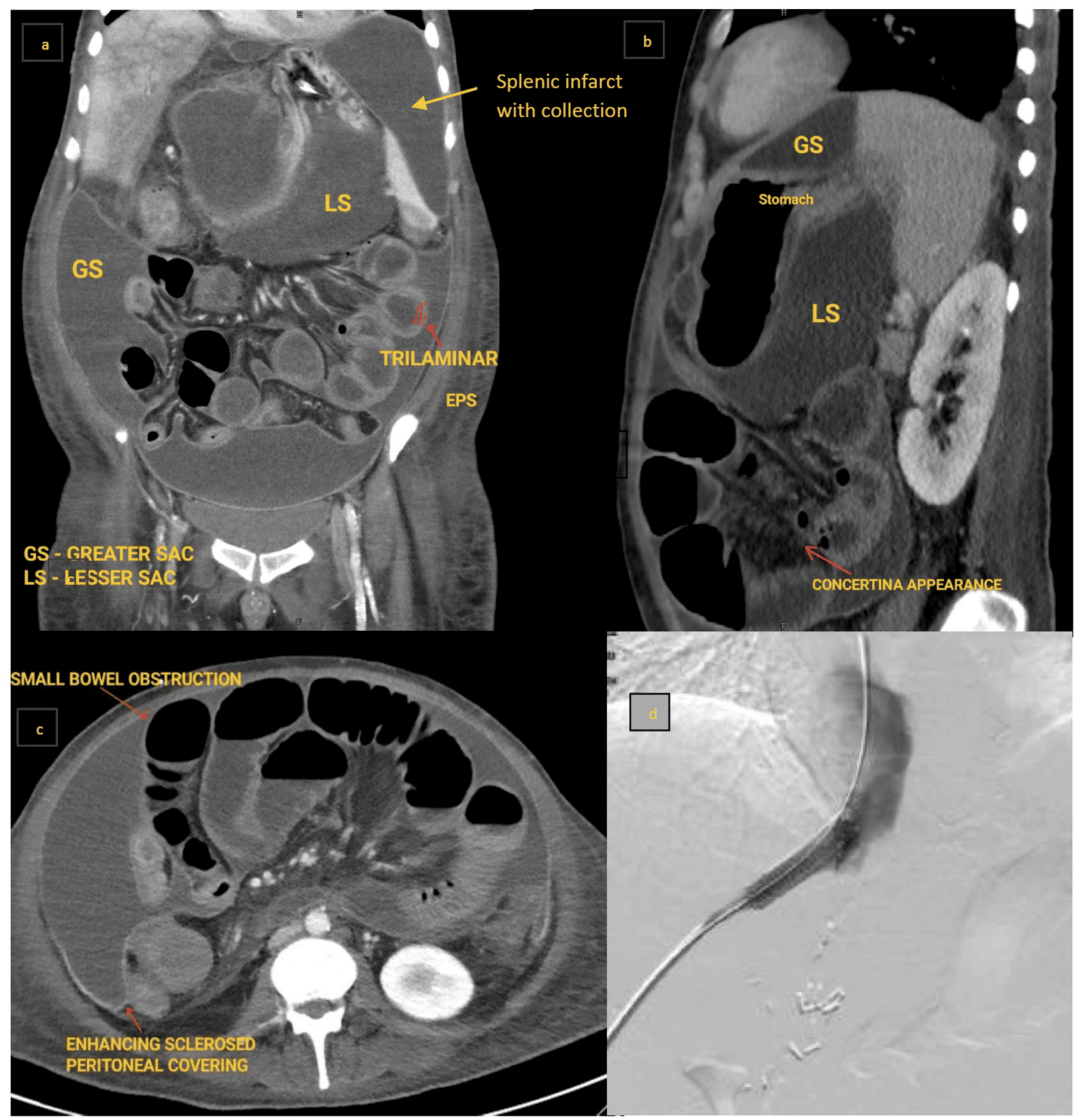
a: Contrast-enhanced CT (coronal) showing encapsulated fluid collections in the greater and lesser sacs, with perisplenic collections due to prior infarction. b: CECT (sagittal) showing encapsulated fluid collection. c: Axial CECT depicting encapsulation around small bowel loops clumped and conglomerated toward the root of the mesentery, with dilatation suggestive of subacute intestinal obstruction. d: Fluoroscopic image post-portal vein stenting. CT: computed tomography, CECT: contrast-enhanced computed tomography, GS: greater sac, LS: lesser sac, EPS: encapsulating peritoneal sclerosis

A 41-year-old male patient with coronary artery disease and type 2 diabetes mellitus, status post-living donor liver transplant (LDLT), and known portal vein thrombosis on oral anticoagulants presented with generalized body pain, abdominal distension, occasional breathing difficulty, and pedal edema for one week. Initial abdominal ultrasound (USG) revealed a thrombus in the portal vein extending to its anterior and posterior branches, as well as middle hepatic vein thrombosis. Portal stenting was performed (Figure 1d). The two-month follow-up ultrasound showed ascites, clumped bowel loops with a trilaminar appearance of a hyperechoic membrane, hypoechoic bowel wall, and hyperechoic bowel lumen/mucosa-probable features of peritoneal encapsulation. Imaging suggested features of encapsulating peritoneal sclerosis (Figure 1a-1c).

The patient was planned for immediate laparotomy and adhesion lysis to relieve peritoneal visceral constriction. Bronchoalveolar lavage (BAL) culture revealed *Stenotrophomonas maltophilia*, with a colony count of >100,000 colony-forming unit (cfu)/mL. The patient succumbed to extensive drug-resistant (XDR) sepsis and multiple organ dysfunction syndrome (MODS).

Case 2

**Figure 2:**
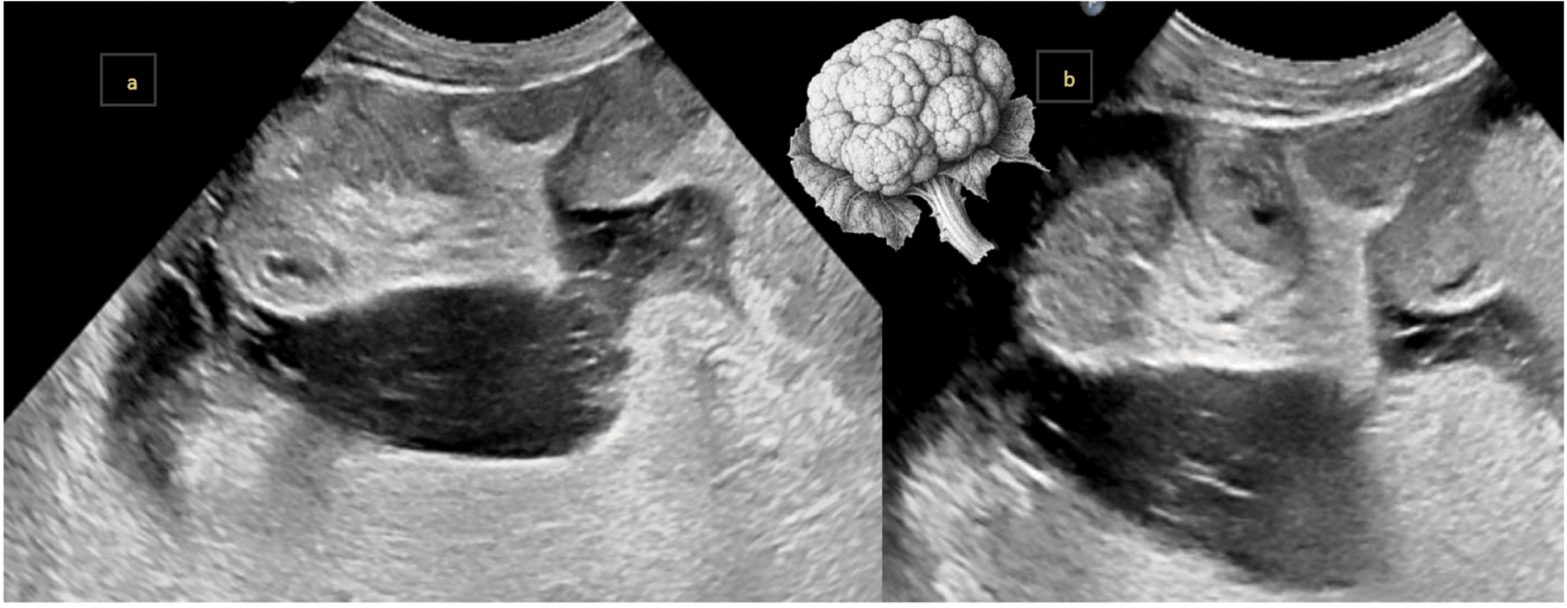
a and b: Abdominal ultrasound images showing central clumping of small bowel loops toward the root of the mesentery, resembling a cauliflower with a trilaminar appearance: hyperechoic membrane, hypoechoic bowel wall, and hyperechoic lumen.

A 31-year-old male patient presented with abdominal distension and weight loss. Ultrasound (USG) findings indicated encapsulating peritoneal sclerosis (EPS), with ascites and clumped small bowel loops (Figure 2a, 2b).

**Figure 3:**
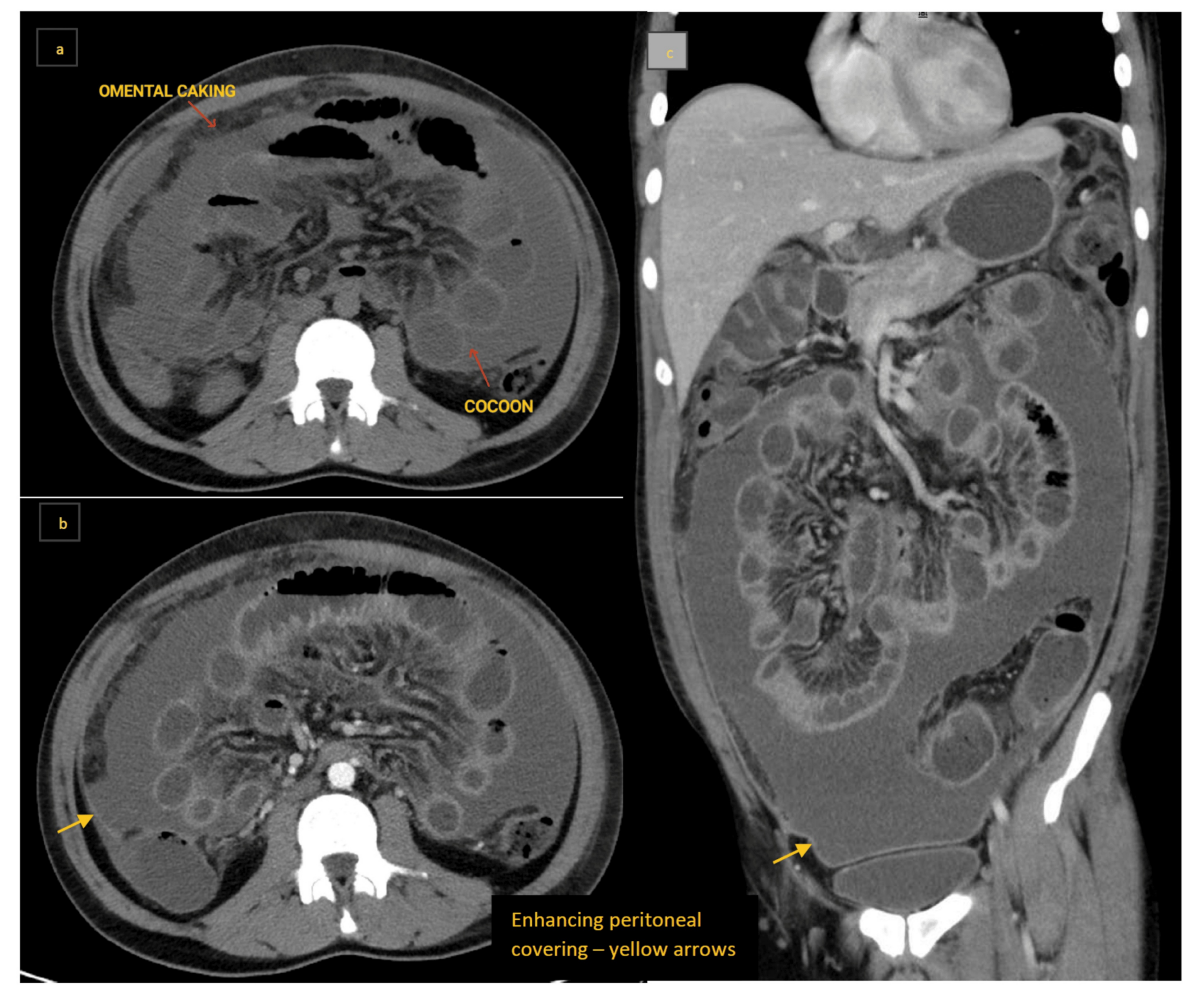
a: Axial non-contrast CT showing encapsulated ascites forming an "abdominal cocoon" with diffuse, smooth omental nodules suggestive of peritoneal TB. b: Axial contrast-enhanced CT with peritoneal enhancement. c: No evidence of proximal bowel dilatation, obstruction, or peritoneal/bowel wall calcifications. CT: computed tomography, TB: tuberculosis

Subsequent contrast-enhanced CT (CECT) confirmed encapsulated ascites and clumped small bowel loops (Figure 3).

Diagnostic laparoscopy and peritoneal/omental biopsy were done, and omental sample histopathology showed a necrotizing (caseating) granulomatous lesion, suggestive of tuberculosis. The acid-fast bacteria (AFB) stain was negative. His GeneXpert *Mycobacterium tuberculosis* (MTB)-rifampicin (RIF) test (peritoneal tissue) revealed a *Mycobacterium tuberculosis* complex.

Antitubercular therapy (ATT) was started, and the patient symptomatically improved and was discharged with advice to be on regular follow-up.

Case 3

A 26-day-old female term neonate with acyanotic congenital heart disease (CHD), type B aortic arch interruption, an aberrant right subclavian artery from the descending thoracic aorta, and small perimembranous ventricular septal defect (VSD) closed by the tricuspid septal leaflet presented with syndromic features possibly indicative of CHARGE syndrome. Findings included right iris coloboma, right corneal opacity, left choanal atresia, facial dysmorphism, and rocker-bottom feet. The neonate developed respiratory distress and desaturation shortly after birth, with notable umbilical cord swelling. Initial abdominal USG and X-ray showed no evidence of perforation or peritonitis, but repeat USG indicated features of bowel obstruction.

**Figure 4:**
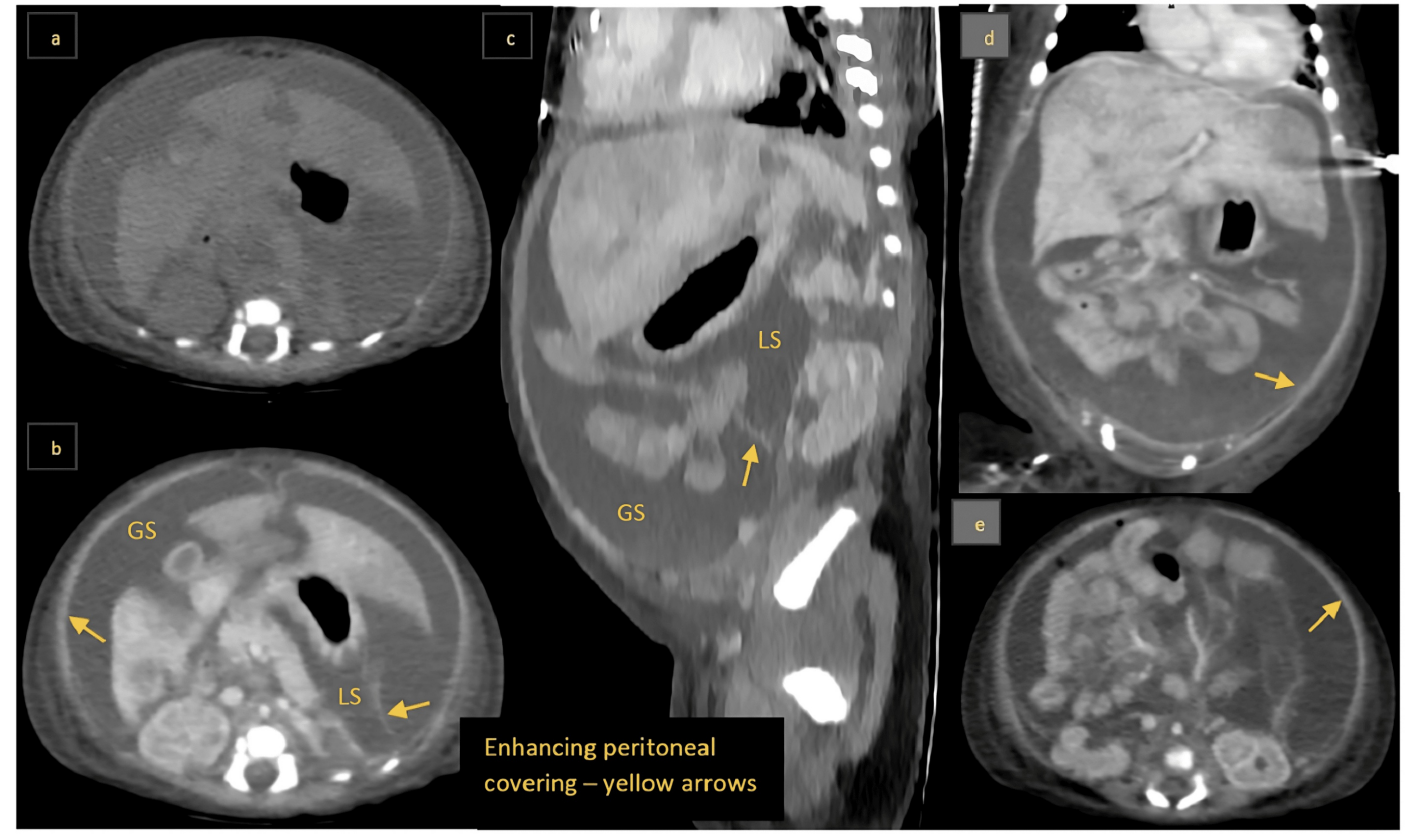
a: Axial non-contrast CT showing gross ascites. b: Axial contrast-enhanced CT showing peritoneal enhancement. c: Sagittal contrast-enhanced CT reconstruction showing loculated ascites in the greater and lesser sacs. d: Coronal contrast-enhanced CT showing clumped small bowel loops with peritoneal enhancement. e: Axial contrast-enhanced CT showing peritoneal encapsulation around the clumped small bowel. CT: computed tomography, GS: greater sac, LS: lesser sac

Contrast-enhanced CT revealed loculated fluid collections in the greater and lesser sacs with enhancing peritoneal covering and small air pockets along the anterior aspect of these collections. Small bowel loops were clumped toward the root of the mesentery without definite dilation, while large bowel loops were collapsed, suggestive of perforation with encapsulated peritonitis. Imaging also showed heterogeneous liver enhancement and communication between the portal vein and inferior vena cava (IVC), likely representing Abernethy malformation (Figure 4). The neonate subsequently experienced cardiopulmonary arrest secondary to septic shock and succumbed.

Case 4

**Figure 5:**
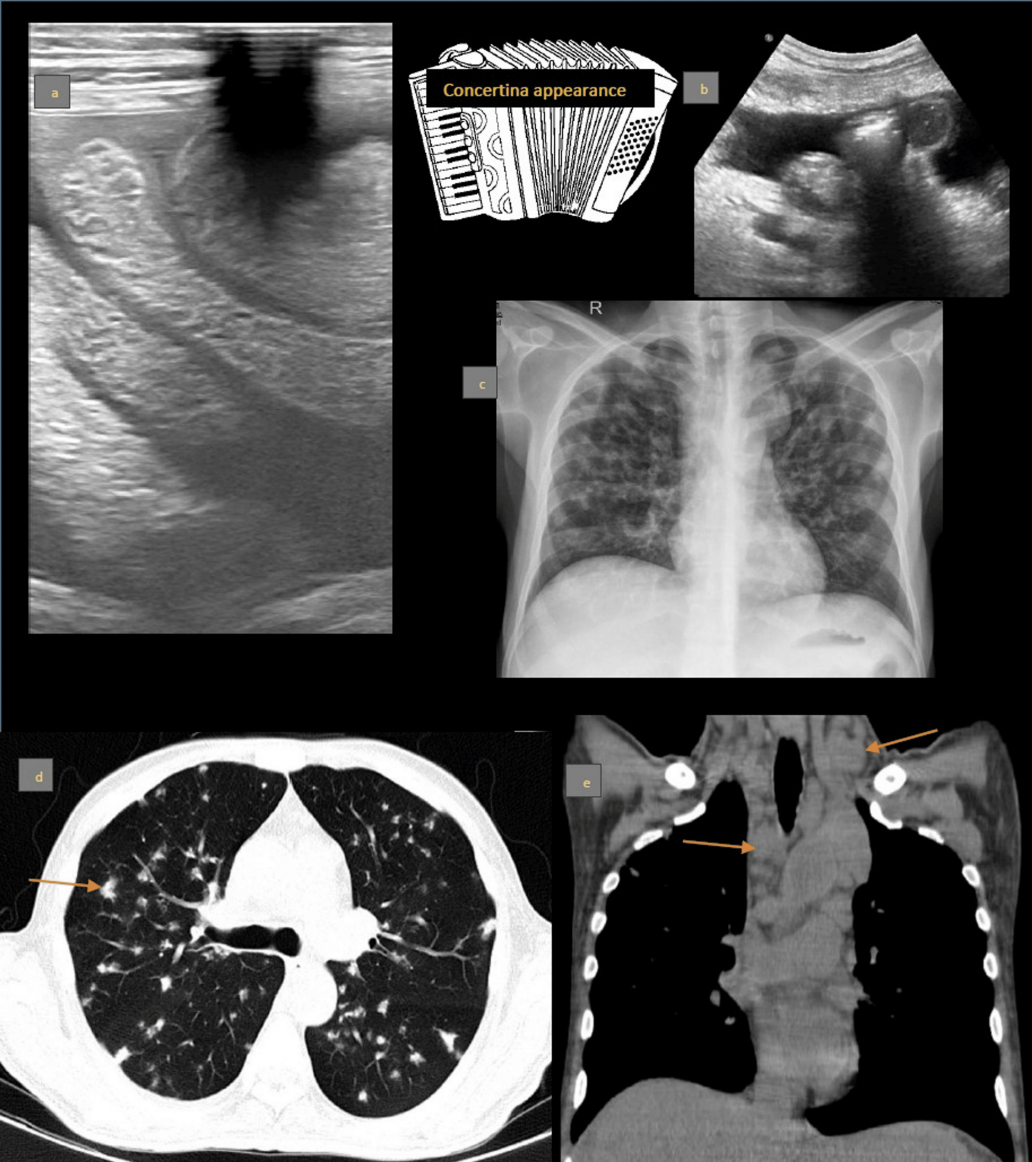
a: Ultrasound showing stacked small bowel loops with a concertina-like appearance. b: Ultrasound showing central clustering of small bowel loops at the mesentery root, resembling a cauliflower. c: Chest radiograph showing patchy nodular opacities in both lungs. d: Axial HRCT showing peribronchovascular nodules. e: Coronal CT of the chest with enlarged mediastinal and neck nodes. HRCT: high-resolution computed tomography, CT: computed tomography

A 41-year-old male patient with diabetes and hypertension presented with a three-week history of fever, two-week history of cough, and one-month history of weight loss (3-4 kg over four months). He was diagnosed with disseminated tuberculosis (TB). Physical examination revealed pallor, abdominal distension, and shifting dullness. Abdominal USG showed liver with altered echotexture, surface irregularities, multiple cysts, and hypoechoic lesions, along with moderate ascites with septations. Small bowel loops were stacked at the root of the mesentery (Figure 5a, 5b). CT of the chest demonstrated endobronchial infection with nodules and peribronchovascular nodular consolidations (Figure 5d, 5e).

**Figure 6:**
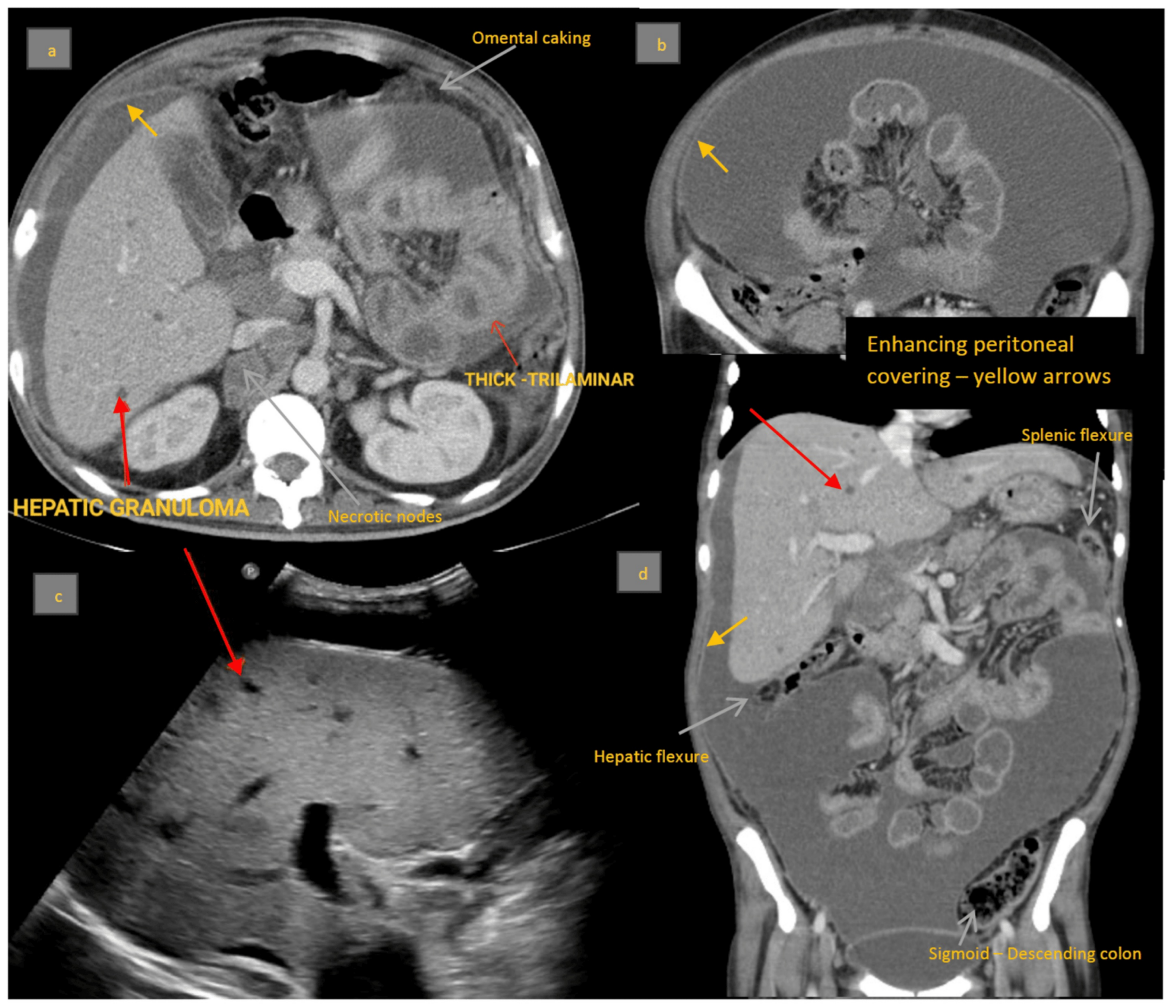
a: Axial post-contrast CT section showing hyperattenuating ascites with diffusely thickened, enhancing peritoneal covering, omental caking, and multiple enlarged and necrotic abdominal lymph nodes. b: Axial post-contrast CT section with central clumping of encapsulated small bowel loops without obstruction. c: Ultrasound of the abdomen showing multiple well-defined hypodense lesions in the liver, likely granulomatous infection. d: Coronal contrast CT section showing loculated ascites with enhancing peritoneal covering suggestive of abdominal cocoon (note the hepatic and splenic flexures and descending colon outside the cocoon). Overall imaging features: wet-type abdominal tuberculosis. CT: computed tomography

Abdominal CT revealed probable peritoneal TB with enhancing peritoneal covering, encapsulating peritoneal sclerosis, multiple hypodense hepatic and splenic lesions, omental caking, and multiple enlarged and necrotic abdominal and mediastinal lymph nodes, suggestive of granulomatous infection (Figure 6a-6d). Overall imaging features suggested wet-type abdominal tuberculosis.

Diagnostic tapping of ascitic fluid revealed low serum-ascites albumin gradient (SAAG), high protein ascites, and elevated adenosine deaminase (ADA) level (92 U/L), suggestive of TB. Ascitic fluid cytopathology showed no atypical or malignant cells, and the fluid culture was negative. Ascitic fluid GeneXpert analysis showed trace positivity. Sputum samples tested negative for Gram staining, AFB, and fungal smear. However, culture revealed Enterobacteriaceae, and antibiotics were continued. Endobronchial ultrasound (EBUS)-guided BAL samples detected *Mycobacterium tuberculosis*, and cytopathology revealed granulomatous lymphadenitis with necrosis. The patient was started on an ATT regimen and showed symptomatic improvement.

## Discussion

Encapsulating peritoneal sclerosis (EPS) is a rare yet significant condition marked by high morbidity and potential mortality. Diagnosing EPS is challenging due to its gradual onset and nonspecific symptoms, which often contribute to diagnostic delays. Radiologic imaging, however, can play an essential role in the early identification of EPS, with timely intervention and potentially improving patient outcomes. Table 2 presents various etiologies for encapsulating peritoneal sclerosis (EPS), highlighting the different underlying conditions that can lead to its development.

**Table 2:** Etiologies of encapsulating peritoneal sclerosis EPS: encapsulating peritoneal sclerosis

Etiologies of EPS	
Primary (idiopathic)	
Secondary: related to peritoneal dialysis [2,19]	Peritoneal dialysis-associated encapsulating peritoneal sclerosis
Secondary: unrelated to peritoneal dialysis	Exposure to abdominal surgery or interventions: laparotomy for carcinoma or benign disorders, abdominal lavage with povidone-iodine, ventriculoperitoneal shunt, LeVeen peritoneovenous shunt; peritonitis: bacterial peritonitis, including tubercular peritonitis and meconium peritonitis; β-blocker administration [3-5]; cirrhosis [6]; bowel perforation [7]; autoimmune diseases: systemic lupus erythematosus [8], peritoneal sarcoidosis [9], and familial Mediterranean fever [10]; after organ transplantation: liver [11], kidney [12], and intestine [13]; malignancies: intra-abdominal (gastric [14], pancreatic, renal, and midgut neuroendocrine), pelvic (ovarian), and lymphoma; intraperitoneal drug administration; radiation enteritis; diseases of the female reproductive tract: luteinized thecoma of the ovary, endometriosis, adenomyosis, leiomyoma, and teratoma

Differential diagnosis

Several conditions fall within the differential diagnosis of EPS, including congenital peritoneal encapsulation, peritoneal carcinomatosis, and internal hernias. Congenital peritoneal encapsulation is a benign condition characterized by a thin accessory peritoneal membrane surrounding the small bowel, typically asymptomatic and usually discovered incidentally during surgery or radiologic examinations. Peritoneal carcinomatosis, on the other hand, is identified by thickening and abnormal enhancement of the peritoneum. It can be differentiated by its nodular thickening pattern and associated findings such as nodules in the omentum, pouch of Douglas, and serosal surfaces, often accompanied by lymphadenopathy and primary tumors such as ovarian or gastric cancers. Internal hernias may also resemble EPS due to the abnormal clustering of bowel loops, but they lack the characteristic soft-tissue mantle of EPS. Additionally, internal hernias tend to occur in fixed anatomical regions and carry a higher risk of bowel ischemia from compromised vascular pedicles [21].

Complications

EPS can lead to severe and sometimes life-threatening complications, significantly impacting patients' quality of life. Bowel obstruction is one of the primary complications, as fibrocollagenous encapsulation of the bowel leads to sclerosis and membrane contraction, mechanically obstructing the bowel. Malnutrition is another significant risk, as recurrent bowel obstructions hinder adequate nutrient absorption. Bowel ischemia and infarction may occur as well, with fibrotic encapsulation compressing bowel loops and compromising the blood supply, potentially leading to ischemia and necrosis. Increased pressure on and weakened integrity of the bowel wall can also result in perforation and subsequent peritonitis, with recurrent infections heightening the risk of systemic sepsis.

Other complications of EPS include extensive adhesions and fistulas between bowel segments or adjacent organs as a result of chronic inflammation and fibrosis. Ascites in EPS patients can further cause respiratory compromise, often necessitating repeated drainage procedures. The fluid imbalance also places patients at risk of electrolyte disturbances and renal dysfunction due to reduced absorption.

Ultimately, EPS is associated with an increased mortality rate, particularly in cases with delayed diagnosis and treatment, underscoring the critical role of radiology in early detection and management. Advanced imaging techniques, including ultrasound, computed tomography (CT), and magnetic resonance imaging (MRI), are invaluable in identifying hallmark features of EPS such as bowel encapsulation, ascites, peritoneal thickening, and the characteristic clumping of bowel loops. Early radiologic identification of these signs allows for timely intervention, which can significantly impact prognosis and reduce the risk of severe complications. Radiology, therefore, plays a pivotal role in guiding clinical decision-making and improving outcomes for patients with EPS through enhanced accuracy and precision in diagnosis.

## Conclusions

Encapsulating peritoneal sclerosis (EPS) is a rare but serious condition often underdiagnosed due to limited awareness among healthcare providers, despite being a leading cause of small bowel obstruction in tertiary care hospitals. Early diagnosis, supported by multimodal imaging such as contrast-enhanced CT (CECT) and ultrasound, is essential for detecting key features and improving patient outcomes. The condition's multifactorial etiology requires individualized treatment plans tailored to the underlying cause.

A collaborative effort among clinicians, radiologists, and researchers is critical to enhance awareness, refine diagnostic approaches, and optimize treatment strategies for EPS. Future research should focus on establishing standardized protocols to improve care and outcomes for patients with this challenging condition.
